# Supplementary material for: Patterns of Intron Gain and Loss in Fungi
Source: PLoS Biol. 2004 Nov 30;2(12):e422. doi: 10.1371/journal.pbio.0020422 (PMC532390; doi:10.1371/journal.pbio.0020422)
Supplement: Table S1 — Also available at http://genes.mit.edu/NielsenEtAl/. (4.3 MB ZIP). [file pbio.0020422.st001.zip › NielsenEtAl/html/1412.html]

AN3928.1.NCU06110.1.MG03098.1.FG02469.1


```
 CLUSTAL W (1.82) Multiple Sequence Alignments - Introns Inserted


Sequence 1: AN3928.1	331 aa
Sequence 2: FG02469.1	322 aa
Sequence 3: NCU06110.1	344 aa
Sequence 4: MG03098.1	327 aa
Alignment Length: 347 aa
Number Identitical Residues: 190 aa
Alignment Score (without introns) 8287


MG03098.1 	MAPPAATSPIASHAHPLDVKN-GKHQHNADSAAAAAAK-LHDLATDWN--QFSFSPIRES
NCU06110.1	MSPSVLEPQSVPTLVNVGLKAVGRNDAPVERDARGLSKPLLELMPTLGTDAFTFSPIRES
FG02469.1 	MSPPAAISP--TSRSVELATSTTKLPVHAVK--NINTKTVEEMLGNWD--DFKFAPIRES
AN3928.1  	MSPPAAIFE--PTVAPTGIKGKVVVPETATIPGDSQTKLLDHFGGKWD--NFKFAPIRES
          	*:*..      .       .        .   .   :* : .:    .   *.*:*****

MG03098.1 	QVSRAMTSRYFADLHAHAETDVVIVGAGSCGLSAAFHLATARPDLRITLVEAGVAPG~GG
NCU06110.1	TVSRAMTRRYFADLDAHAETDIVIVGAGSCGLSAAYVLSTLRPDLRITIVEAGVAPG~GG
FG02469.1 	QVSRAMTRRYFQDLDNYAESDIVIIGAGSCGLSAAYILGKKRPDLKIAIIEASVSPG~GG
AN3928.1  	QVSRAMTRRYFQDLDRYAESDVVIVGAGSCGLSTAYVLAKARPDLKIAIIEASVSPG1GG
          	 ****** *** **. :**:*:**:********:*: *.. ****:*:::**.*:** **

MG03098.1 	AWLGGQLFSAMVMRKPAHLFLDRLGVAYEDE---GSFVVVKHAALFTSTLLSKVLALDNV
NCU06110.1	AWLGGQLFSAMVMRKPADVFLDEVGVPYEDE---GDYVVVKHAALFTSTVLSKVLQRPNV
FG02469.1 	AWLGGQLFSAMIMRKPADAFLREVGVPYEDE---GNYVVVKHAALFTSTIMSKVLQMPNI
AN3928.1  	AWLGGQLFSAMVMRRPAELFLNELGVPYEEDPDMPNYVVVKHASLFTSTLLSKVLSFPNV
          	***********:**:**. ** .:**.**::..  .:******:*****::****   *:

MG03098.1 	KLFNATAVEDLITRR------------EGGDNAGVRVAGVVTNWTLVSMHHDDQSCMDPN
NCU06110.1	KLFNATTVEDLITRKHHAESSSSSDDGEAEDEAKVRIAGVVTNWTLVSMHHDDQSCMDPN
FG02469.1 	KLFNATCVEDLITRP-----------SDEG----IRIAGVVTNWTLVSMHHDDQSCMDPN
AN3928.1  	KLFNATCVEDLVTRP-----------GPNGNAQEVQIAGVVTNWTLVTLHHDDHSCMDPN
          	****** ****:**            .   .   :::**********::****:******

MG03098.1 	TINAPVVISTTGHDG1PFGAFSAKRLVSMKQIEQLGGMRGLDMQSAEDAIVKRTREIVPG
NCU06110.1	TINAPVIISTTGHDG~PFGAFSVKRLVSMKQMERLNGMRGLDMQSAEDAIVNNTREIVPG
FG02469.1 	TINAPLIISTTGHDG~PMGAFCVKRLVSMQRIEKLGGMRGLDMNLAEDAIVKGTREIVPG
AN3928.1  	TINAPVIISTTGHDG~PFGAFSAKRLVSMTTIDKLGGMRGLDMNSAEDAIVKNTREVAKG
          	*****::******** *:***..******  :::*.*******: ******: ***:. *

MG03098.1 	LIIGGMELSEIDGANRMG1PTFGAMVLSGVKAAEEAMAVYDTRKKQNAY-
NCU06110.1	LIVGGMELSEIDGANRMG1PTFGAMALSGVKAAHEAIRVFDLRKAQNDKC
FG02469.1 	LIVGGMELSEVDGANRMG1PTFGAMALSGLKAAEEALKIFDTRKKQNEQ-
AN3928.1  	LIIGGMELSEIDGFNRMG~PTFGAMVLSGVKAAEEALRVFDDRKRECAE-
          	**:*******:** **** ******.***:***.**: ::* ** :
```
